# Comparative Analysis of Dementia Care Programs and Delivery Models

**DOI:** 10.1093/geroni/igab046.549

**Published:** 2021-12-17

**Authors:** Allie Peckham, Marianne Saragosa, Madeline King, Monika Roerig, Gregory Marchildon

**Affiliations:** 1 Arizona State University, Pheonix, Arizona, United States; 2 University of Torono, Toronto, Ontario, Canada; 3 University of Toronto, Toronto, Ontario, Canada

## Abstract

Dementia has significant social and economic impacts for those living with dementia and their caregivers. Despite an increase in prevalence of complex chronic conditions and dementia, long-term care services are continuously pushed out of institutional settings and into the home and community. The majority of people living with dementia in Canada and the United States (U.S.) live at home with support provided by family, friends, or other unpaid caregivers. Ten dementia care programs and service delivery models across five different North American jurisdictions in Canada and the U.S. are compared using a deductive analytical approach using a comparative policy framework developed by Richard Rose. The policy efforts included in this research attempt to improve health system flow and access for vulnerable populations. One common theme among all jurisdictions are long-standing institutional barriers that can make change difficult. These barriers can prevent the ability for systems to be flexible and adapt to meet the changing needs of populations. Incrementalism is often considered an appropriate approach to health system reform. Yet, incremental change efforts lead to policy layers and these layers can lead to tension between different policy mixes and unintended consequences. These programs were introduced in a manner that did not fully consider how to patch current structures and risk creating further system redundancies. One approach to reduce this risk is to combine evaluative efforts that assess ‘goodness of fit’. The degree to which these programs have embedded these efforts successfully is low, with the possible exception of DSRIP from NY.

